# The Effect of Rutin and Extracts of *Uncaria guianensis* (Aubl.) J. F. Gmeland on Primary Endometriotic Cells: A 2D and 3D Study

**DOI:** 10.3390/molecules25061325

**Published:** 2020-03-13

**Authors:** Camila Hernandes, Renata Nascimento de Oliveira, Artur Henrique de Souza Santos, Helena Malvezzi, Bruna Cestari de Azevedo, Bárbara Yasmin Gueuvoghlanian-Silva, Ana Maria Soares Pereira, Sergio Podgaec

**Affiliations:** 1 Hospital Israelita Albert Einstein, Av. Albert Einstein 627, Morumbi 05651-901, São Paulo, SP, Brazil; renata.nascimento9292@gmail.com (R.N.d.O.); arturhenriquesantos@gmail.com (A.H.d.S.S.); helena.malvezzi@einstein.br (H.M.); bruna.cestari@einstein.br (B.C.d.A.); barbara.ygs@gmail.com (B.Y.G.-S.); sergiopodgaec@me.com (S.P.); 2 Universidade de Ribeirão Preto, Av. Costabile Romano 2201, Ribeirania 14096-900, Ribeirão Preto, SP, Brazil; apereira@unaerp.br; 3 Departamento de Obstetrícia e Ginecologia, Faculdade de Medicina, Universidade de São Paulo, Av. Dr. Arnaldo 455, Cerqueira César 01246-903, São Paulo, SP, Brazil

**Keywords:** endometriosis, ROS production, anti-inflammatory, anti-oxidant, cytokines, growth factors, medicinal plants

## Abstract

There is increasing interest in the potential of natural compounds to treat diseases, such as endometriosis, a gynecological disorder that affects 10–15% of women of reproductive age, and it is related to severe pelvic pain and infertility. We have evaluated the in vitro effects of rutin and the aqueous bark, roots, and leaf extracts (ABE, ARE, and ALE, respectively) and isolated components of *Uncaria guianensis* on stromal cells from eutopic endometrium and lesions of patients with endometriosis. Two- and three-dimensional cultures were used to assess the cell death and production of reactive oxygen species (ROS), cytokines and growth factors of cells following exposure to these natural products. The applied treatments did not reduce cellular viability, but ROS production did increase. In addition, significant increases in the levels of interleukin (IL)-15, IL-17A, IL-4, IL-6, tumor necrosis factor-α, and vascular endothelium growth factor were observed when 2D-cells from endometrium of patients with endometriosis were treated with ABE, while exposure to ALE induced significant increases in epidermal growth factor in lesion cells.

## 1. Introduction

Endometriosis is a gynecological disorder that affects 10–15% of women of reproductive age, with up to 70% of patients reporting pelvic pain and 48% presenting infertility [1,2]. The disease is characterized by the presence of endometrial stromal and/or glandular epithelial cells outside the uterus and can be classified into three different forms: superficial, ovarian, and deep endometriosis [2,3,4]. Although the condition has been known since the 17th century and has received considerable research effort over the past 30 years, the pathogenesis of the disease is not yet clear [5,6]. The theory most accept is the one proposed by Sampson [7], of retrograde menstruation, which describes that the foci of endometriosis originate from the adhesion of menstrual endometrial tissue in the peritoneal cavity and other organs, resulting from retrograde tubal flow. But it is known that factors like anatomical, genetic, endocrine, environmental, oxidative stress, and inflammatory reactions are involved in this process, since although 90% of women have retrograde menstruation, but only 10% develop the disease [8,9,10,11].

The disorder is often associated with dysmenorrhea, dyspareunia, acyclic pelvic pain, and infertility [12,13,14], symptoms that can be debilitating and affect quality of life [15]. The treatment of endometriosis aims to alleviate pelvic pain and restore fertility, and may involve drug therapy or surgery depending on the clinical characteristics of the patient and the disease. 

Medication is the primary approach for women with pelvic pain. Progesterones, with or without estrogens, are the first-line therapy for endometriosis since they promote pelvic pain relief in about 70% of patients [16]. However, the use of progestogens may be accompanied by side-effects such as amenorrhea, escape bleeding, acne, weight gain, and undesirable contraceptive effects in women who wish to conceive and symptoms of endometriosis tend to resume when treatment is discontinued, [17,18].

In the absence of improvement after clinical treatment or in the case of obstructive intestinal lesions, surgical intervention is the alternative option [2], but drug treatment should be maintained over an extended period, even after improvement of symptoms, in order to prevent recurrence of clinical manifestations [17,18].

As an alternative to hormone therapy, administration of non-steroidal anti-inflammatory drugs often produces satisfactory results, but these medications offer only symptomatic relief and are associated with undesirable side effects such as gastric ulcers [17].

Considering the lack of a fully effective drug therapy for endometriosis, the search for non-hormonal therapeutic agents has increased, and ideally, such long-term medication, with better curative clinical efficacy should involve administration of a low-cost, easy-to-use therapeutic agent with minimal adverse effects, which some natural compounds are promising candidates.

In light of the above, which we hypothesized that the use of plant-derived anti-oxidants would improve the levels of biomarkers relating to pain, inflammation, and oxidative stress associated with endometriosis. In order to test this hypothesis, we assessed the effect of pure rutin as well as aqueous extracts of *U. guianensis* and their active components (mitraphylline, isomitraphylline, quinic, and chlorogenic acids) on the viability, susceptibility and levels of ROS, cytokine, and growth factors using two- and three-dimensional (2D and 3D, respectively) culture models of primary stromal cells isolated from patients with and without endometriosis. The choice of primary culture cells, as well as the comparison of two culture models, may facilitate the discovery of new drugs for the treatment of endometriosis.

The glycosylated flavonoid rutin possesses potent anti-oxidant and anti-inflammatory activities and plays an important role in the regulation of ROS production, while the herbal remedies *Uncaria guianensis* (Aubl.) J. F. Gmel. (Rubiaceae), and *U. tomentosa* (Willd. ex Schult.) DC., commonly known as “unha-de-gato” (cat’s claw), have been used in traditional medicine to treat inflammatory diseases [19,20].

## 2. Results

### 2.1. Identification of Alkaloids and Phenolics in Extracts of U. guianensis 

**Figure 1:**
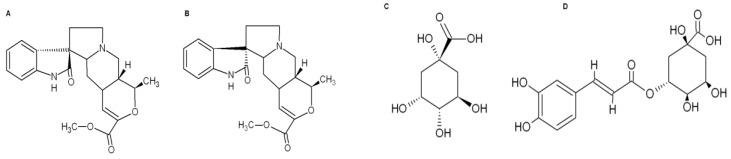
Chemical structures of the active principles of *Uncaria guianensis* extracts: (**A**) mitraphylline, (**B**) isomitraphylline, (**C**) quinic acid, and (**D**) chlorogenic acid.

The pentacyclic oxindole alkaloids mitraphylline and isomitraphylline along with the phenolic compounds quinic and chlorogenic acids (Figure 1A–D) were identified by high pressure liquid chromatography (HPLC) in aqueous leaf, bark, and root extracts (ALE, ABE, and ARE, respectively) from *U. guianensis* (Supplementary Figures S1 and S2). As shown in Table 1, the levels of the oxindole alkaloids differed among the extracts with ALE presenting the highest concentrations.

### 2.2. Viability of Endometrial Stromal Cells

**Figure 2:**
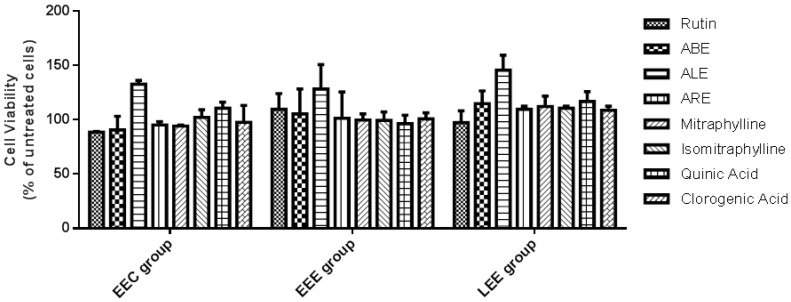
Within-group comparisons of viabilities of stromal cells isolated from eutopic endometrium of control patients without endometriosis (EEC group), eutopic endometrium of patients with endometriosis/endometrioma (EEE group), and lesions of patients with endometriosis/endometrioma (LEE group) after treatment with rutin, aqueous bark extract (ABE), aqueous leaf extract (ALE), or aqueous root extract (ARE) from *Uncaria guianensis* at 1000 µg mL^−1^, or with mitraphylline, isomitraphylline, quinic acid, or chlorogenic acid at 5 µg mL^−1^. Assays were carried out using a 2D culture technique and the MTT method. Each bar represents mean percentage viability ± standard deviation (*n* = 3) in relation to untreated cells (100% viability). Data were analyzed by one-way ANOVA and Tukey test with no statistical significance set at *p* > 0.05.

The effects of rutin, ALE, ABE, ARE, mitraphylline, isomitraphylline, quinic, and chlorogenic acids on the viability of stromal cells originating from eutopic endometrium of control patients without endometriosis (EEC group), eutopic endometrium of patients with endometriosis/endometrioma (EEE group), and lesions of patients with endometriosis/endometrioma (LEE group) were evaluated using a two-dimensional (2D) culture model and the 3-(4,5-dimethylthiazole-2-yl)-2,5-diphenyltetrazolium bromide (MTT) method. Within-group comparisons showed that after 24 h of treatment there were no significant differences between the viabilities of treated EEC, EEE, and LEE cells compared with untreated cells (Figure 2).

### 2.3. Susceptibility of Endometrial Stromal Cells

**Figure 3:**
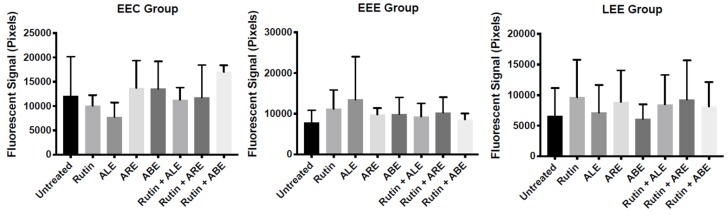
Within-group comparisons of the susceptibilities of stromal spheroids isolated from eutopic endometrium of control patients without endometriosis (EEC group), eutopic endometrium of patients with endometriosis/endometrioma (EEE group), and lesions of patients with endometriosis/endometrioma (LEE group) to treatment with rutin, aqueous bark extract (ABE), aqueous leaf extract (ALE), or aqueous root extract (ARE) from *Uncaria guianensis* at 100 µg mL^−1^, or with combinations of rutin + ALE, rutin + ABE, and rutin + ARE containing 100 μg mL^−1^ of each component. Assays were carried out using a 3D cell technique and SYTOX™ Green nucleic acid stain, while untreated spheroids served as control. Each bar represents the mean fluorescence in pixels ± standard deviation (*n* = 3). Data were analyzed by one-way ANOVA and Tukey test with statistical significance set at *p* < 0.05.

The susceptibilities of spheroids from the EEC, EEE, and LEE groups towards rutin, ALE, ABE, ARE, rutin + ALE, rutin + ABE, and rutin + ARE were evaluated using a three-dimensional (3D) culture model and SYTOX™ Green nucleic acid staining. Within-group comparisons showed that there were no significant differences between treated EEC, EEE, and LEE spheroids and their untreated counterparts (Figure 3) with respect to cell death. In contrast, between-group comparisons revealed that, in comparison with EEC spheroids, there were significant reductions in fluorescent intensities of EEE and LEE spheroids treated with rutin + ABE suggesting that these spheroids were more resistant to treatment-induced cell death (Supplementary Figure S3). Comparisons of spheroids from eutopic endometrium and lesions of patients with retrocervical endometriosis (RE subgroup) and ovarian endometrioma (OE subgroup) demonstrated that susceptibilities to treatments were similar regardless of the type of endometriosis (Supplementary Figure S4).

### 2.4. Production of Reactive Oxygen Species (ROS) in Endometrial Stromal Cells

**Figure 4:**
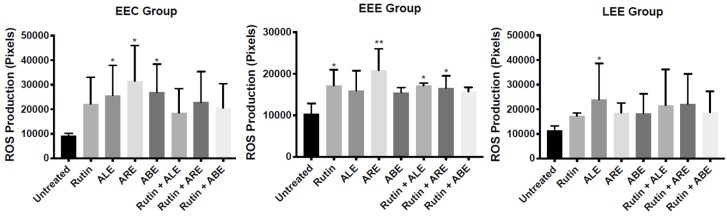
Within-group comparisons of reactive oxygen species (ROS) production by stromal cells isolated from eutopic endometrium of control patients without endometriosis (EEC group), eutopic endometrium of patients with endometriosis/endometrioma (EEE group), and lesions of patients with endometriosis/endometrioma (LEE group) following treatment with rutin, aqueous bark extract (ABE), aqueous leaf extract (ALE), or aqueous root extract (ARE) from *Uncaria guianensis* at 100 µg mL^−1^, or with combinations of rutin + ALE, rutin + ABE, and rutin + ARE containing 100 μg mL^−1^ of each component. Assays were carried out using a 2D cell technique with dichlorodihydrofluorescein diacetate as probe, while untreated cells served as control. Each bar represents the mean number of pixels ± standard deviation (*n* = 3). Data were analyzed by one-way ANOVA and Tukey test with statistical significance set at *p* < 0.05. (* *p* < 0.05; **p < 0.001).

The production of ROS by EEC, EEE, and LEE cells treated with rutin, ALE, ABE, ARE, rutin + ALE, rutin + ABE, and rutin + ARE was evaluated using a 2D culture model and dichlorodihydrofluorescein diacetate as probe. In comparison with untreated cells, levels of ROS in EEC group cells treated with ALE, ABE, and ARE increased significantly (*p* < 0.05), while in the EEE group ROS increased significantly (*p* < 0.05 and 0.001) in cells treated with rutin, ARE, rutin + ALE, and rutin + ARE. Within the LEE group, the concentration of ROS increased significantly (*p* < 0.05) in comparison with controls only in cells treated with ALE (Figure 4). Between-group comparisons revealed no statistically significant differences in ROS production among EEC, EEE, and LEE group cells regardless of the treatment (Supplementary Figure S5). 

**Figure 5:**
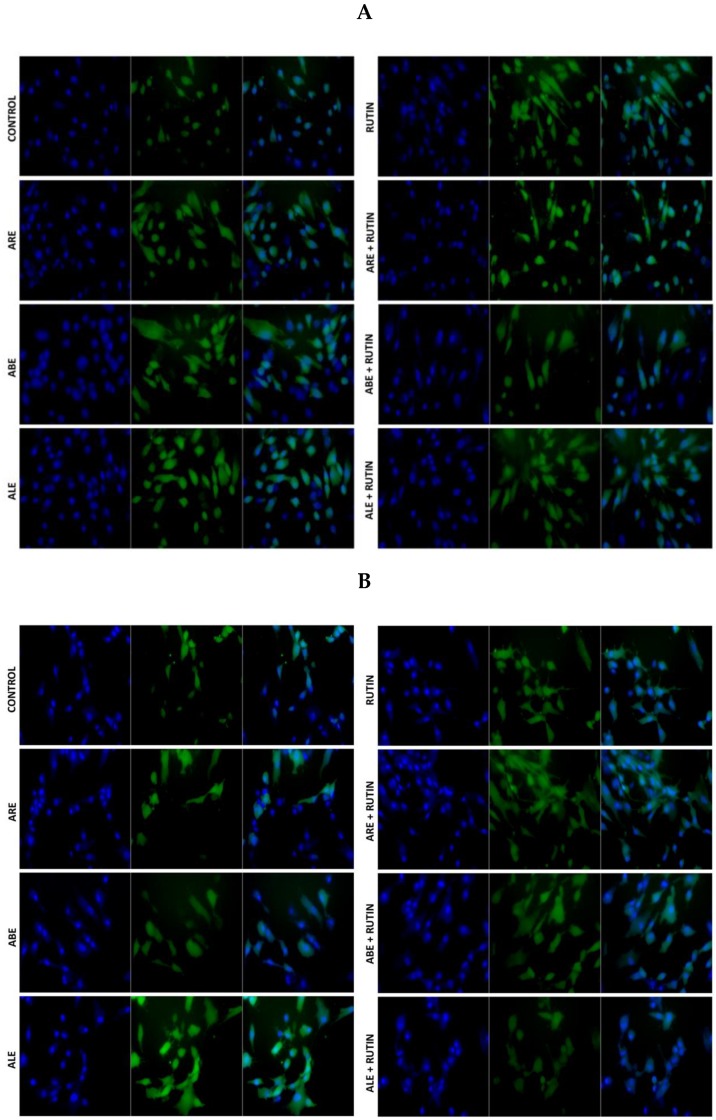
Fluorescent signals indicating the production of reactive oxygen species (ROS) by (**A**) eutopic endometrium-derived stromal cells and (**B**) lesion-derived stromal cells collected from an individual patient with retrocervical endometriosis (RE subgroup). Cells were treated with Rutin, aqueous bark extract (ABE), aqueous leaf extract (ALE), or aqueous root extract (ARE) from *Uncaria guianensis* at 100 µg mL^−1^, or with combinations of Rutin + ALE, Rutin + ABE, and Rutin + ARE containing 100 μg mL^−1^ of each component. Assays were carried out using a 2D cell technique with dichlorodihydrofluorescein diacetate as probe, while untreated cells served as control. Images were acquired and analyzed at 20× magnification with a Carl Zeiss Axio Vert.A1 microscope running Zen 2010 software.

Comparisons between cells obtained from eutopic endometrium and lesions of patients with retrocervical endometriosis (RE subgroup) and ovarian endometrioma (OE subgroup) revealed that the production of ROS was similar regardless of the type of endometriosis or the treatment applied (Supplementary Figure S6). Figure 5 shows the intensities of fluorescent signals emitted after exposure to the various treatments by eutopic endometrium-derived and lesion-derived stromal cells collected from an individual patient in the RE subgroup.

**Figure 6:**
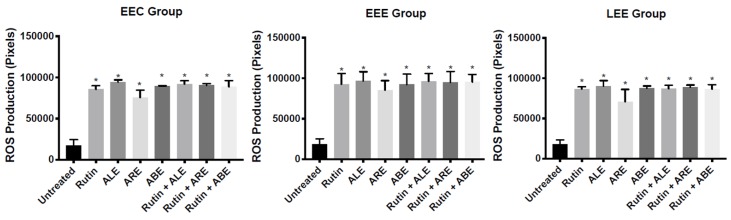
Within-group comparisons of reactive oxygen species (ROS) production by stromal spheroids isolated from eutopic endometrium of control patients without endometriosis (EEC group), eutopic endometrium of patients with endometriosis/endometrioma (EEE group), and lesions of patients with endometriosis/endometrioma (LEE group) following treatment with rutin, aqueous bark extract (ABE), aqueous leaf extract (ALE), or aqueous root extract (ARE) from *Uncaria guianensis* at 100 µg mL^−1^, or with combinations of rutin + ALE, rutin + ABE, and rutin + ARE containing 100 μg mL^−1^ of each component. Assays were carried out using a 3D cell technique with the dichlorodihydrofluorescein diacetate as probe, while untreated spheroids served as control. Each bar represents the mean number of pixels ± standard deviation (*n* = 3). Data were analyzed by one-way ANOVA and Tukey test with statistical significance set at * *p* < 0.0001.

The effects of rutin, ALE, ABE, ARE, rutin + ALE, rutin + ABE, and rutin + ARE on ROS production by EEC, EEE, and LEE spheroids were evaluated using a 3D culture model. The production of ROS in all three groups was significantly augmented (*p* < 0.0001) by the treatments in comparison with untreated spheroids (Figure 6). In contrast, between-group comparisons revealed no statistically significant differences among the groups regardless of the treatment (Supplementary Figure S7).

**Figure 7:**
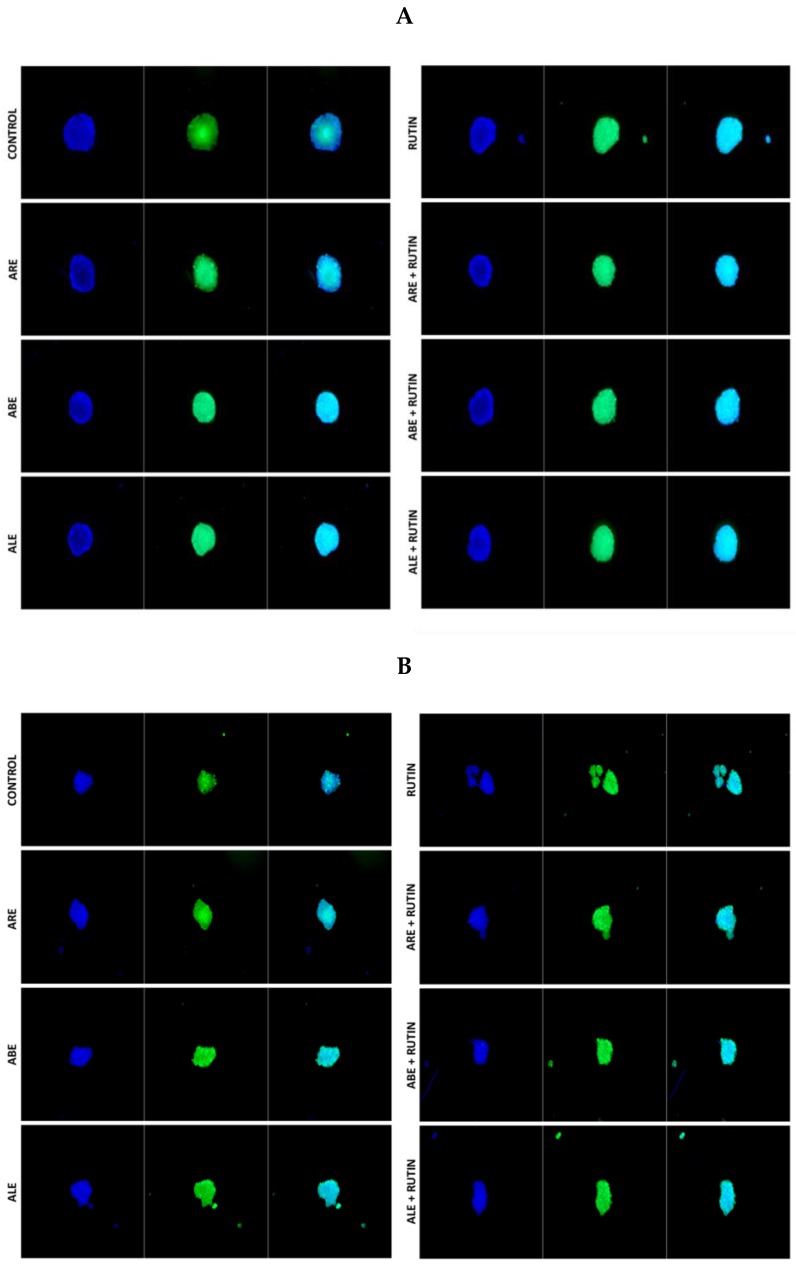
Fluorescent signals indicating the production of reactive oxygen species (ROS) by (**A**) eutopic endometrium-derived stromal spheroids and (**B**) lesion-derived stromal spheroids collected from an individual patient with retrocervical endometriosis (RE subgroup). Spheroids were treated with rutin, aqueous bark extract (ABE), aqueous leaf extract (ALE), or aqueous root extract (ARE) from *Uncaria guianensis* at 100 µg mL^−1^, or with combinations of rutin + ALE, rutin + ABE, and rutin + ARE containing 100 μg mL^−1^ of each component. Assays were carried out using a 3D cell technique with dichlorodihydrofluorescein diacetate as probe, while untreated spheroids served as control. Images were acquired and analyzed at 20× magnification with a Carl Zeiss Axio Vert.A1 microscope running Zen 2010 software.

Comparisons between spheroids obtained from eutopic endometrium and lesions of patients with retrocervical endometriosis (RE subgroup) and ovarian endometrioma (OE subgroup) revealed that ROS production was similar, regardless of the type of endometriosis (Supplementary Figure S8). Figure 7 shows the intensities of fluorescent signals emitted after exposure to the various treatments by eutopic endometrium-derived and lesion-derived stromal spheroids collected from an individual patient in the RE subgroup. 

### 2.5. Production of Inflammatory Markers in Endometrial Stromal Cells

The levels of cytokines and growth factors in untreated EEE and LEE cells and those treated with rutin, ALE, ABE, and ARE at 10 or 100 μg mL^−1^ were evaluated using 2D and 3D culture techniques. When the 2D cell model was employed, EEE cells treated with ABE at 100 µg mL^−1^ exhibited significantly higher (*p* < 0.05) levels of interleukin 15 (IL-15), IL-17A, IL-4, IL-6, tumor necrosis factor-alpha (TNF-α), and vascular endothelium growth factor (VEGF) in comparison with untreated cells. In addition, LEE cells treated with ALE at 100 µg mL^−1^ showed significantly higher levels of epidermal growth factor (EGF) compared with untreated cells (Table 2). No significant differences were observed in the levels of these analytes in EEE and LEE cells exposed to the other treatments (Supplementary Tables S1 to S7). Regarding the levels of the other cytokines and growth factors assessed, namely IL-10, IL-1A, IL-1B, IL-2, IL-8, transforming growth factor alpha (TGF-α), and TNF-β, no significant differences were observed between untreated and treated EEE and LEE cells (data not shown). When the 3D cell model was employed, the levels of all 14 analytes tested remained unchanged in both EEE and LEE spheroids exposed to the treatments in comparison with untreated cells (data not shown).

## 3. Discussion

We have evaluated the effects of rutin, aqueous extracts of *U. guianensis* (alone and in combination with rutin), and the purified active principles (mitraphylline, isomitraphylline, quinic, and chlorogenic acids) on endometrium-derived and lesion-derived stromal cells collected from patients with endometriosis in order to test the hypothesis that these natural products would improve the levels of biomarkers of pain, oxidative stress and inflammation associated with this disorder. 

Various proposals have been put forward to explain how endometriosis develops, but the most widely accepted hypothesis is that the retrograde flow of menstrual contents reaches the ovary and surrounding tissues via the fallopian tubes [7]. Regardless of the etiopathogenesis of the disease, it is known that hormonal and inflammatory regulatory factors are involved in the development and maintenance of lesions [21,22]. It has also been postulated that a deficient immune response could prevent the immune system from removing endometrial cells present in the retrograde menstrual flow [23]. Furthermore, the peritoneal cavity of patients with endometriosis is a proactive environment with a high capacity for the production of ROS, and this may contribute to the maintenance of oxidative stress and inflammatory reaction associated with the disease [24]. Considering that the inequality between levels of ROS and anti-oxidants is a key element in the pathogenesis of endometriosis [25], therapies that aim to reduce oxidative imbalance would be expected to alleviate the symptoms of the condition, as stated in our original hypothesis. 

The glycosylated flavonoid rutin was selected for study because of its recognized anti-oxidant [26,27,28], anti-inflammatory [29,30,31], and anti-angiogenic [32] properties. The anti-inflammatory activities of *Uncaria* spp. have been previously demonstrated [19,20,33], thereby justifying the study of extracts and pure compounds derived from *U. guianensis* with the aim of improving the clinical manifestations of endometriosis. The chemical profiles of aqueous extracts of *U. guianensis* established herein were similar to those reported by Sandoval et al. [20] and characterized by low concentrations of pentacyclic oxindole alkaloids and high concentrations of phenolic compounds. 

Within-group comparisons established that the viabilities and susceptibilities of EEC, EEE, and LEE cells that had been treated with rutin, extracts of *U. guianensis*, either alone or in combination with rutin, or the active principles of *U. guianensis*, were similar to those of untreated cells. However, between-group comparisons revealed that the levels of SYTOX dead-cell stain fluorescence in EEE and LEE cells that had been treated with rutin + ABE were significantly reduced in comparison with those of EEC cells exposed to the same treatment, indicating that EEE and LEE cells are more resistant to treatment-induced cell death. These results are in accord with a recent report from our research group [34] in which we describe reduced levels of a lamin B1, a protein responsible for cellular apoptosis and senescence, and the presence of apoptosis-resistant cells in endometrial lesions of patients with endometriosis. Since the regulation of the apoptotic process is defective in cells of endometrial lesions, such tissues may not respond to drugs that normally induce cell death in other tissues [23].

The anti-oxidant effects of the natural products studied were evaluated using 2D and 3D culture techniques. While traditional 2D cell cultures are the mainstay of biological research, 3D spheroid cultures present structural and functional characteristics that mimic in vivo cells and their environment [35] and the results of assays performed in the two culture systems may differ. Indeed, when we assessed the effects of rutin, ALE, ABE, and ARE, either alone or in combination, on EEC, EEE, and LEE cells, some of the treatments increased ROS production in 2D cultures (Figure 4), whereas all of the treatments augmented ROS production in 3D cultures (Figure 6). 

Interestingly, we observed no differences in the basal production of ROS between endometrium of control patients and eutopic endometrium or lesions of patients with endometriosis in either the 2D or 3D culture systems. This finding is not in accord with literature data indicating that endometriotic cells are more oxidized [8,25]. Moreover, Chen et al. [36] reported that ectopic endometrial mitochondria from patients with ovarian endometrioma generated more ROS and energy than eutopic endometrial mitochondria from the same patients or from control patients without endometriosis. In addition, Ngô et al. [37] reported that cells from ectopic endometrium of patients with ovarian endometrioma displayed increased ROS production and altered ROS detoxification pathways that were correlated with increased cellular proliferation and activation of extracellular signal–regulated kinases (ERKs). It is possible, however, that this phenomenon was occasioned by changes in cell metabolism under different culture conditions.

Regarding the levels of inflammatory markers, EEE cells grown in 2D cultures and treated with ABE displayed increased production of the pro-inflammatory factors IL-6, IL-15, IL-17A, and TNF-α, together with the anti-inflammatory IL-4 and VEGF, in comparison with untreated cells. In addition, LEE cells also exhibited increased levels of EGF when exposed to ALE. However, no alterations in the levels of cytokines or growth factors were observed in 3D cultures of stromal cells. There is ample evidence to suggest that endometriosis is associated with aberrant immune and inflammatory responses characterized by high levels of pro-inflammatory cytokines [6,10,23,38,39,40,41,42]. It appears, therefore, that ABE potentiates the already intense inflammatory response of endometriotic tissues by activating signaling pathways that regulate inflammatory mediator levels. 

The results presented herein showed that rutin, aqueous extracts from *U. guianensis* extracts and their combinations were not able to control oxidative stress and inflammation when applied to stromal cells from endometrium and lesions of patients with endometriosis. Our findings differ from those of Sandoval et al. [20] who reported that extracts of *U. guianensis* and *U. tomentosa* provide effective anti-oxidant and anti-inflammatory activities, with the former being more potent. According to these authors, the concentration of alkaloids in *U. tomentosa* is 35-fold higher than that in *U. guianensis*, signifying that the level of alkaloids did not influence the anti-oxidant and anti-inflammatory activity of these species. 

The increased production of ROS observed in EEE and LEE cells exposed to rutin, *U. guianensis* extracts and their combinations leads us to speculate that a longer treatment time would produce an increase in cell death. Such a possibility exists because, along with the reported increase in ROS accumulation in endometriotic tissues, it is known that the production of ROS is directly linked to the process of apoptosis. For example, it has been shown that some phenolic compounds, including the flavonoid naringenin, act as pro-oxidants and stimulate ROS production leading to the induction of apoptosis of endometrial cells after 24 h exposure with collapse of mitochondrial membranes, increased mitochondrial ROS, inhibition of anti-apoptotic Bcl-2 proteins and activation of caspase-3 [43]. In this context, Ferella et al. [44] established that the *O*-methylated flavone wogonin increased ROS accumulation but inhibited cell proliferation and induced cell cycle arrest in in vitro cultures of human endometrial stromal cells, and reduced lesion size while increasing the percentage of apoptotic cells in endometriotic-like lesions in an in vivo mouse model of endometriosis. In addition, Park et al. [45,46] reported that apigenin exhibited anti-proliferative and apoptotic activities towards human endometriosis cell lines while luteolin displayed similar properties not only in vitro but also in an in vivo mouse model of endometriosis, and that the effects of both flavonoids were accompanied by dose-dependent increases in ROS accumulation. Although rutin belongs to the same chemical class as luteolin and apigenin, treatment of EEE and LEE cells with this flavonoid did not reduce viability nor did it induce cell death despite the augmentation of ROS. Furthermore, treatment of EEE and LEE cells with *U. guianensis* extracts ABE, ALE, and ARE (alone or in combination with rutin) did not increase cell death, different from our previous observations with *U. tomentosa.* The anti-inflammatory properties of the active principles of *Uncaria* species have been tested previously on macrophages and tumor cells [19,20] but not on endometriotic cells, and this may explain the conflicting results presented herein.

When 2D cultures of LEE cells were treated with ALE, the levels of EGF increased concomitantly with the rise in ROS production. EGF is a chemo-attractant protein that induces the growth, proliferation, and migration of epidermal and epithelial cells, including endometrial cells, through the activation of the extracellular signal-regulated kinase 1/2 (ERK1/2) signaling pathway [47,48,49] and high levels of this factor may not be helpful in the case of endometriosis. Of particular note is the observation by Rakhila et al. [49] that EGF levels in the peritoneal fluid of endometriotic patients were markedly higher than those of the controls.

The most important group of secondary metabolites found in *U. guianensis* and *U. tomentosa* are the oxindole alkaloids mitraphylline, isomitraphylline, rhinchophylline, isorinchophylline, pteropodine, isopteropodine and uncarin F [50,51], with the first two compounds being considered chemical markers of the species [52]. Environmental factors exert a strong influence on the chemical profile of a medicinal plant, while the pharmacological activities of extracts depend in part on synergism between the bioactive constituents [53,54,55] and, for these reasons, standardization of herbal products is of the utmost importance. It is meaningful to note that *U. guianensis* is widely distributed [56] and variations in the amounts of active components may have contributed for the differential biological effects of the extracts and their ineffectiveness in inducing the death of endometriotic cells. 

There are still relatively few experimental studies for new endometriosis treatments involving natural products, and this still use immortalized cells, and in a two-dimensional model.

It is be important to validate models for these tests, and for this reason, this work has brought about findings in both two-dimensional and three-dimensional culture models, once as proved in this work, there is a difference between the results obtained in different cell model, and that these differences may result in contradictions in studies carried out in animals and in humans.

Despite the practicality and low cost of two-dimensional cultures, this model does not mimic the disposition of cells in endometriotic lesions, nor does it represent cell–cell interactions and the cell–extracellular environment. Factors such as cell differentiation, proliferation, and gene and protein expression, are affected and can influence the ability to respond to drug metabolism, in addition to other cellular functions [57].

Therapies against undesirable forms of cell proliferation, as in cancer or endometriosis, often function through induction of apoptosis and therapy failure may be explained, in some cases, by cell resistance to apoptosis. The treatments applied in the present study were not able to induce death in stromal cells from eutopic endometrium or lesions from patients with endometriosis or to reduce the production of ROS and inflammatory mediators in these cells as we originally hypothesized. Nevertheless, the results of this study have provided some insights for future research, namely: (i) 2D and 3D culture models yielded divergent outcomes, most likely associated with the distinct microenvironments of cells and spheroids, hence raising the question of reliability of models employed in endometriosis experiments; (ii) the 2D cell model indicated that the levels of some inflammatory markers were up-regulated by 100 μg mL^−1^ of ABE or ALE and this could have therapeutic significance. For instance, the ABE-induced upregulation of IL-15 is particularly interesting since this protein enhances the anti-tumor activity of natural killer (NK) cells by potentiating NKG2D receptors and is a possible candidate molecule for anti-tumor therapy. 

A longer time of exposure of the cells to the treatments with the assets may have been a major limitation of the study, and further studies should be carried out. Another important factor is endometriotic lesions are composed of stromal and epithelial cells. This study evaluated only the effect on stromal cells.

## 4. Material and Methods 

### 4.1. Preparation of Aqueous Extracts of Uncaria guianensis

Plants of *U. guianensis* were collected with permission from the National System of Genetic Resource Management and Associated Traditional Knowledge (SisGen), the Genetic Heritage Management Council (CGen) and the Brazilian Ministry of Environment, and a voucher specimen was deposited in the Herbarium of Medicinal Plants of the University of Ribeirao Preto with registration code AC9FFCB. Leaves, bark and roots were separated, dried in a circulating air oven at 45 °C for 3 days and ground in a Marconi (Piracicaba, SP, Brazil) MA048 knife mill fitted with a 45 mesh sieve. Samples (50 g) of each of type of plant tissue were extracted separately with 1 L of water at 100 °C for 20 min by infusion (leaves) or decoction (bark and roots), filtered through filter paper and the filtrates reduced to dryness. The residues were labeled aqueous leaf extract (ALE), aqueous bark extract (ABE), or aqueous root extract (ARE), as appropriate, and stored at 4 °C until required for assay. 

### 4.2. Extraction and Quantification of Mitraphylline and Isomitraphylline

The pentacyclic oxindole alkaloids mitraphylline and isomitraphylline were extracted from *U. guianensis* following the procedure described by Bertol et al. [58]. Leaves, bark and roots from individual specimens were dried and reduced to a fine powder (as described in Section 4.1) and samples (100 mg) submitted to static maceration with 1 mL aliquots of methanol in amber flasks at room temperature (25 °C) for 24 h. The mixtures were filtered and the filtrates reduced to dryness in a fume cupboard. All extractions were performed in triplicate. 

Prior to analysis by HPLC, extracts were submitted to a solid-phase extraction (SPE) clean-up procedure on Supelco-57054 C18 cartridges (Sigma Aldrich, St. Louis, MO, United States) that had been previously eluted with 1 mL of methanol (J.T. Baker HPLC grade; Avantor Performance Materials, Center Valley, PA, USA) followed by 1 mL of an 80:20 (*v/v*) mixture of methanol and Milli-Q Ultrapure water (Merck Millipore, Darmstadt, Germany). Dried extracts (15 mg) were redissolved in 1 mL of 80:20 (*v/v*) methanol:water mixture and applied to SPE cartridges that were subsequently eluted with 3 mL of the methanol:water mixture. Aliquots (20 μL) of eluents (5 mg mL^−1^) were analyzed on a Shimadzu (Kyoto, Japan) model LC-10ADvp instrument coupled to a Shimadzu SPD-M10Avp photo diode array (PDA) detector and fitted with a Zorbax Eclipse XDB-C18 column (150 × 4.6 mm i.d., 5 μm; Agilent, Santa Clara, CA, USA) protected by a Zorbax Eclipse XDB-C18 pre-column (4.6 × 12.5 mm i.d., 5 μm). Gradient elution was performed at room temperature (22 °C) with acetonitrile (solvent A; J.T. Baker HPLC grade) and 10 mmol L^−1^ aqueous ammonium acetate adjusted to pH 6.9 with triethanolamine (solvent B; Neon, Sao Paulo, Brazil) supplied at a flow rate of 0.8 mL min^−1^ according to the program: 35% A between 0 and 18 min, 50% A between 18 and 25 min, 35% to 100% A from 25 and 40 min, and finally 35% A between 40 and 45 min. The detection wavelength was set at 245 nm and the acquired data were processed using Shimadzu LabSolutions Multi LC-PDA software.

Alkaloid content was determined using a previously validated HPLC-PDA method [59] with mitraphylline and isomitraphylline (CromaDex, Los Angeles, CA, USA; product numbers ASB 00013955-005 and ASB-00009417-005, respectively) as external standards. Linearity, precision, accuracy, limit of detection (LoD) and limit of quantification (LoQ) of the analysis were established according to the guidelines of the Brazilian National Health Surveillance Agency [60]. Respective LoD and LoQ values were 0.22 and 0.75 μg mL^−1^ for mitraphylline and 0.12 and 0.24 μg mL^−1^ for isomitraphylline. Calibration curves were constructed using standard alkaloids at concentrations of 7.8, 15.6, 31.25, 62.5, 125, and 200 μg mL^−1^ with each concentration injected in triplicate. The peak area ratios of mitraphylline and isomitraphylline were calculated and plotted against the corresponding standard concentrations using linear regression of the standard curves.

### 4.3. Analysis of Quinic and Chlorogenic Acids in Aqueous Extracts from U. guianensis

The presence of quinic and chlorogenic acids in ALE, ABE, and ARE was confirmed by ultra-performance liquid chromatography-mass spectrometry (UPLC-MS) using the chromatographic set up described in Section 4.2, operated in the negative ion mode. Aliquots (5 µL) of extracts and reference standards (1 mg mL^−1^) were injected onto an Ascentis Express C18 column (100 × 4.6 mm i.d.; 2.7 µm; Sigma Aldrich) and gradient eluted with a mixture of formic acid (0.1%) in methanol (solvent A) and formic acid (0.1%) in water (solvent B) supplied at a flow rate of 0.5 mL min^−1^ according to the program: 3% A between 0 and 4 min, 3% to 60% A between 4 and 15 min, 60% to 90% A between 15 and 19 min, and finally 3% A between 19 and 24 min. The source was maintained at 150 °C, the capillary voltage was 2.5 kV, the desolvation temperature was 300 °C, the flow rate of desolvation gas (N_2_) was 600 L h^−1^, and the mass scan range was 100–700 *m/z* in the full scan mode. All analyses were carried out in triplicate.

### 4.4. Patients and Sampling

The study was conducted in accordance with the rules of the Declaration of Helsinki of 1975 (revised in 2013), and the protocol was approved on May, 2017 by the Research Ethics Committee of Hospital Israelita Albert Einstein (project number 66505217.4.0000.0071). Written informed consent was obtained from all participants prior to the collection of tissues. Samples of eutopic endometrium and lesions were collected from patients who had been referred to laparoscopic surgery for treatment of retrocervical endometriosis (*n* = 2) or ovarian endometrioma (*n* = 2). Additional samples of endometrium were collected from patients without endometriosis (*n* = 2) who had been referred to laparoscopy for treatment of other diseases such as uterine fibroids and benign ovarian cysts. None of the participants received hormonal treatment prior to surgery. 

### 4.5. Isolation and Culture of Primary Stromal Cells from Eutopic Endometrium and Lesions

Tissues collected from the eutopic endometrium (EEE group) and lesions (LEE group) of patients with retrocervical endometriosis (subgroups RE) or ovarian endometrioma (subgroups OE), and from the eutopic endometrium (EEC group) of control patients without endometriosis, were transferred immediately to phosphate-buffered saline containing 1% antibiotic-antimycotic solution (Thermo Fisher Scientific, Waltham, MA, USA). Primary stromal and epithelial cell cultures were established as previously described [61,62]. Briefly, tissues were cut into small fragments with a scalpel and digested with type IV collagenase (1.0 mg mL^−1^) for 90 min at 37 °C. Stromal cells were separated from tissue debris and glandular tubules by filtering the digested cell suspension through 250- and 35-μm cell strainers. The filtrate containing stromal cells was centrifuged and the sediment resuspended in a 1:1 (*v/v*) mixture of Dulbecco’s modified Eagle’s medium and F12 nutrient mix (DMEM/F12; Thermo Fisher Scientific) supplemented with 10% fetal bovine serum (SFB) and 1% antibiotic-antimycotic solution. The cells were seeded into culture bottles containing DMEM/F12/SFB medium supplemented with antibiotic/antimycotic solution and incubated in a 5% CO_2_ incubator at 37 °C. After reaching 80% confluence at the fourth passage, the cells were trypsinized, counted and resuspended in fresh cryopreservation medium containing SFB and 10% dimethyl sulfoxide (DMSO). The cells were subsequently frozen by gradually decreasing the temperature initially to −20 °C and then to −80 °C and finally stored in liquid nitrogen. 

### 4.6. Cell Characterization

Cells of the EEE, LEE, and EEC groups that had been grown to the fourth passage were thawed, transferred to DMEM/F12/SFB medium under the conditions stated in Section 4.5, trypsinized and seeded in 25-well pre-filled imaging plates with coverslips at a concentration of 25,000 cells well^−1^. After 24 h, the cells were fixed with 4% paraformaldehyde solution in PBS. The purities of isolated epithelial and stromal cells were determined immunocytochemically by labeling with DAKO^®^ (Agilent Dako, Santa Clara, CA, USA) primary monoclonal antibody, anti-vimentin at 1:100 (*v/v*) dilution followed by a fluorescein isothiocyanate (FITC) conjugated secondary antibody. Images were obtained using a Carl Zeiss (Oberkochen, Germany) model LSM 710 confocal microscope. 3T3 fibroblasts cells were used as positive controls for vimentin staining.

### 4.7. Assessment of Cell Viability Using a 2D Culture Model 

The viabilities of EEE, LEE, and EEC cells that had been treated with rutin, ALE, ABE, and ARE at 1000 μg mL^−1^ in PBS, or mitraphylline, isomitraphylline, quinic and chlorogenic acids at 5 μg mL^−1^ in PBS, were evaluated using a 2D culture technique with untreated cells as controls. Cells that had been stored in liquid nitrogen were thawed and cultured in bottles containing DMEM/F12/SFB medium supplemented with antibiotic/antimycotic solution until they reached confluence, following which they were trypsinized, resuspended in fresh culture medium and seeded at a concentration of 5000 cells well^−1^ in Costar^®^ 96-well 2D culture plates (Thermo Fisher Scientific). Twenty-four hours after plating, cells were exposed to treatment and incubated for a further 24 h in a 5% CO_2_ incubator at 37 °C. Subsequently, the medium was removed, aliquots (100 µL) of 3-(4,5-dimethyl-thiazol-2-yl)-2,5-diphenyltetrazolium bromide (MTT; Sigma-Aldrich) in culture medium (0.5 mg mL^−1^) were added to all wells, including those containing untreated cells, and the plates were incubated for 4 h as before. After incubation, the cells were centrifuged at 2000 rpm for 5 min, the culture medium was removed and aliquots (100 µL) of DMSO were added to the wells and the plates incubated for 1 h as before. The reduction of MTT salt (yellow) to formazan (purple) was determined colorimetrically by reading the absorbance at 550 nm using a SpectraMax i3x microplate reader (Molecular Devices, San Jose, CA, USA). The results were expressed as mean percentage viability ± standard deviation of triplicate assays in relation to untreated cells (100% viability).

### 4.8. Assessment of Cell Susceptibility Using a 3D Culture Model

The susceptibilities of EEE, LEE, and EEC spheroids treated with rutin, ALE, ABE of ARE at 100 μg mL^−1^ in PBS, or combinations of rutin + ALE, rutin + ABE, and rutin + ARE containing 100 μg mL^−1^ of each component, were evaluated using a 3D culture technique with untreated spheroids as controls. Cells that had been stored in liquid nitrogen were thawed, cultured, trypsinized, and resuspended in fresh culture medium as described in Section 4.7, following which they were seeded at a concentration of 5000 cells well^−1^ in Nuclon™ Sphera™ 96-well 3D plates (Thermo Fisher Scientific) to promote the formation of spheroids. Plates were incubated in a 5% CO_2_ incubator at 37 °C for 24 h, and the spheroids evaluated using an Olympus (Tokyo, Japan) model FSX100 inverted microscope before being exposed to treatment and incubated for a further 24 h as before. Cellular death was determined using SYTOX™ Green nucleic acid stain (Thermo Fisher Scientific), according to the manufacturer’s instructions, which penetrates disrupted membranes that are typical of dead cellular material. Stained spheroids were examined under a Zeiss LSM 710 confocal microscope fitted with Zen 2010 software for image analysis, and the fluorescent signal was measured using ImageJ^®^ software. The results were expressed as mean pixels ± standard deviation of triplicate assays.

### 4.9. Quantification of ROS Using 2D and 3D Culture Models

The production of ROS by EEE, LEE, and EEC cells/spheroids following treatment as described in Section 4.8, was evaluated using 2D and 3D culture techniques with untreated cells/spheroids as controls. Cells that had been stored in liquid nitrogen were thawed, cultured, trypsinized, and resuspended in fresh culture medium as described in Section 4.7, after which they were seeded at a concentration of 5000 cells well^−1^ either in Costar® 96-well 2D culture plates or in Nuclon™ Sphera™ 96-well 3D plates, and incubated in a 5% CO_2_ incubator at 37 °C and 90% relative humidity for 24 h. Cells/spheroids were treated with 100 µM of the ROS indicator 2′,7′-dichlorodihydrofluorescein diacetate (Thermo Fisher Scientific) and incubated for 30 min as before. The indicator solution was subsequently removed and the cells/spheroids were incubated in PBS for a further 30 min, after which the PBS was removed and the cells/spheroids exposed to treatment and incubated for 30 min. Cells/spheroids were examined under an Axio Vert.A1 microscope (Carl Zeiss) fitted with Zen 2010 software for image analyses and the results expressed as pixels ± standard deviation of triplicate assays.

### 4.10. Quantification of Inflammatory Markers Using 2D and 3D Culture Models 

The levels of cytokines and growth factors in EEE and LEE cells/spheroids treated with rutin, ALE, ABE, and ARE at 10 or 100 μg mL^−1^ were evaluated using 2D and 3D culture techniques with untreated cells/spheroids as controls. Cells/spheroids, prepared as described in Section 4.9, were exposed to treatment and incubated in a 5% CO_2_ incubator at 37 °C and 90% relative humidity for 24 h, following which the supernatants were collected and stored at –80 °C until required for assay. Analyses were carried out using Milliplex® MAP Human Cytokine/Chemokine Magnetic Bead Panel - Immunology Multiplex Assay (Merck, Darmstadt, Germany) according to the manufacturer´s instructions. The analytes included in the kit were EGF, VEGF, TGF-α, TNF-α, TNF-β, IL-1A, IL-1B, IL-2, IL-4, IL-6, IL-8, IL-10, IL-15, and IL-17A. Readings were performed using a Luminex® multiplexing instrument (Merck) and the results expressed as mean concentrations (pg mL^−1^) and 95% CI. 

### 4.11. Statistical Analyses 

Within-group (untreated × treated cells) and between-group (EEC × EEE ×LEE) comparisons of data relating to viability, susceptibility and ROS production were performed using one-way ANOVA (with or without Welch-correction as appropriate) followed by Tukey or Dunnet post-hoc multicomparison tests. Comparisons between RE and OE subgroups were carried out using two-way ANOVA. All analyses were performed using GraphPad Prism 6.0 software (San Diego, CA, USA) with the statistical significance of the differences set at *p* < 0.05.

For inflammatory markers, the levels of analytes in each treatment were evaluated considering the values obtained from cells of the same patient. Data were modeled using generalized linear mixed models [63], which include normal and gamma distributions, as well as identity and log link functions. The best-fit model was selected on the basis of the entry/exit Aikake Information Criteria (AIC). The results of the final model were presented as mean values, 95% confidence intervals and *p*-values. In multi-comparisons (untreated × treated groups), the *p*-values were corrected using sequential Bonferroni procedure. All analyses were performed using SPSS software with the level of statistical significance set at *p* < 0.05.

## Figures and Tables

**Table 1:** Concentrations of pentacyclic oxindole alkaloids present in aqueous extracts from *Uncaria guianensis.*

Extract	Mitraphylline (mg g^−1^ Dry Weight)	Isomitraphylline (mg g^−1^ Dry Weight)
ALE	0.10 ± 0.010	0.15 ± 0.020
ABE	< LoD and LoQ	0.06 ± 0.003
ARE	< LoD and LoQ	0.03 ± 0.001

ALE, aqueous leaf extract; ABE, aqueous bark extract; ARE, aqueous root extract; LoD, limit of detection; LoQ, limit of quantification.

**Table 2:** Variation in the levels of cytokines and growth factors determined using Milliplex® MAP Human Cytokine/Chemokine Magnetic Bead Panel in 2D cultured stromal cells isolated from eutopic endometrium (EEE group) or lesions (LEE group) of patients with aqueous bark extract (ABE) or aqueous leaf extract (ALE) from *Uncaria guianensis*.

Analyte	Estimated Mean pg mL^−1^ (95% CI)	Estimated Mean Difference ^1^ pg mL^−1^ (95% CI)	*p*-Value *
**IL-15**			
Untreated EEE cells	0.56 (0.26, 1.23)	-	-
ABE (100 μg mL^−1^) treated EEE cells	1.72 (0.97, 3.06)	1.16 (0.04, 2.28)	0.038
**IL-17A**			
Untreated EEE cells	0.14 (−0.10, 0.38)	-	-
ABE (100 μg mL^−1^) treated EEE cells	0.63 (0.39, 0.86)	0.48 (0.16, 0.81)	0.001
**IL-4**			
Untreated EEE cells	2.71 (0.83, 8.86)	-	-
ABE (100 μg mL^−1^) treated EEE cells	13.25 (8.20, 21.41)	10.55 (1.65, 19.44)	0.013
**IL-6**			
Untreated EEE cells	62.21 (−4.17, 128.58)	-	-
ABE (100 μg mL^−1^) treated EEE cells	161.68 (95.31, 228.05)	99.48 (47.71, 155.25)	<0.001
**TNF-α**			
Untreated EEE cells	1.00 (−1.06, 3.06)	-	-
ABE (100 μg mL^−1^) treated EEE cells	3.75 (1.69, 5.81)	2.75 (0.29, 5.20)	0.021
**VEGF**			
Untreated EEE cells	0.00 (−4.55, 4.55)	-	-
ABE (100 μg mL^−1^) treated EEE cells	11.08 (6.53, 15.63)	11.08 (2.99, 19.18)	0.003
**EGF**			
Untreated LEE cells	3.52 (1.52, 5.53)	-	-
ALE (100 μg mL^−1^) treated LEE cells	7.07 (5.06, 9.07)	3.54 (0.85, 6.24)	0.005

CI, confidence interval; IL, interleukin; TNF, tumor necrosis factor; VEGF, vascular endothelium growth factor; EGF, epidermal growth factor. ^1^ In relation to untreated cells. * Statistically significant at *p* < 0.05.

## Supplementary Materials

The following are available online. Figure S1: Ultra performance liquid chromatography- mass spectrometry (UPLC-MS) spectra of *Uncaria guianensis* extracts showing peaks corresponding to the oxindole alkaloids, Figure S2: UPLC-MS spectra of *U. guianensis* extracts showing peaks corresponding to the phenolic compounds, Figure S3: Between-group comparisons showing the intensities of fluorescence of SYTOX™ Green nucleic acid stain indicating dead stromal spheroids isolated from eutopic endometrium of control patients without endometriosis (EEC group), eutopic endometrium of patients with endometriosis/endometrioma (EEE group) and lesions of patients with endometriosis/endometrioma (LEE group). Figure S4: Comparisons of intensities of fluorescence of SYTOX™ Green nucleic acid stain indicating dead stromal spheroids isolated from eutopic endometrium and lesions of patients with retrocervical endometriosis (RE subgroup) and ovarian endometrioma (OE subgroup), Figure S5: Between-group comparisons showing the intensities of fluorescence of dichlorodihydrofluorescein diacetate probe indicating production of reactive oxygen species (ROS) by stromal cells isolated from eutopic endometrium of patients in the EEC, EEE, and LEE groups, Figure S6: Comparisons of intensities of fluorescence of dichlorodihydrofluorescein diacetate probe indicating production of ROS by stromal cells isolated from eutopic endometrium and lesions of patients in the RE and OE subgroups, Figure S7: Between-group comparisons showing the intensities of fluorescence of dichlorodihydrofluorescein diacetate probe indicating production of ROS by stromal spheroids isolated from eutopic endometrium of patients in the EEC, EEE, and LEE groups, Figure S8: Comparisons of intensities of fluorescence of dichlorodihydrofluorescein diacetate probe indicating production of ROS by stromal spheroids isolated from eutopic endometrium and lesions of patients in the RE and OE subgroups. Tables S1–S7: Levels of interleukin-15, IL-17A, IL-4, IL-6, tumor necrosis factor-α, vascular endothelium growth factor, and epidermal growth factor, respectively, determined using Milliplex® MAP Human Cytokine/Chemokine Magnetic Bead Panel in 2D cultured stromal cells isolated from eutopic endometrium of patients in the EEE group and treated with aqueous extracts of *U. guianensis* or rutin.
