# Supplementary material for: The Effect of Rutin and Extracts of Uncaria guianensis (Aubl.) J. F. Gmeland on Primary Endometriotic Cells: A 2D and 3D Study
Source: Molecules. 2020 Mar 13;25(6):1325. doi: 10.3390/molecules25061325 (PMC7144928; doi:10.3390/molecules25061325)
Supplement: Supplementary file 1 [file molecules-25-01325-s001.pdf]

Supplementary Material

The effect of rutin and extracts of *Uncaria guianensis* (Aubl.) J. F. Gmeland on primary endometriotic cells : a 2D and 3D study

Camila Hernandes [1,*], Renata Nascimento de Oliveira [1], Artur Henrique de Souza Santos [1], Helena Malvezzi [1], Bruna Cestari de Azevedo [1], Barbara Yasmin Gueuvoghlanian-Silva [1], Ana Maria Soares Pereira [2] and Sergio Podgaec [1, 3]

1 Hospital Israelita Albert Einstein, Av. Albert Einstein 627, Morumbi, 05651-901, São Paulo, SP, Brazil
2 Universidade de Ribeirão Preto, Av. Costabile Romano 2201, Ribeirania, 14096-900, Ribeirão Preto, SP, Brazil
3 Departamento de Obstetrícia e Ginecologia, Faculdade de Medicina, Universidade de São Paulo, Av. Dr. Arnaldo 455, Cerqueira César, 01246-903, São Paulo, SP, Brazil
* Correspondence: camila.hernandes@einstein.br; Tel: +55-11-2151031

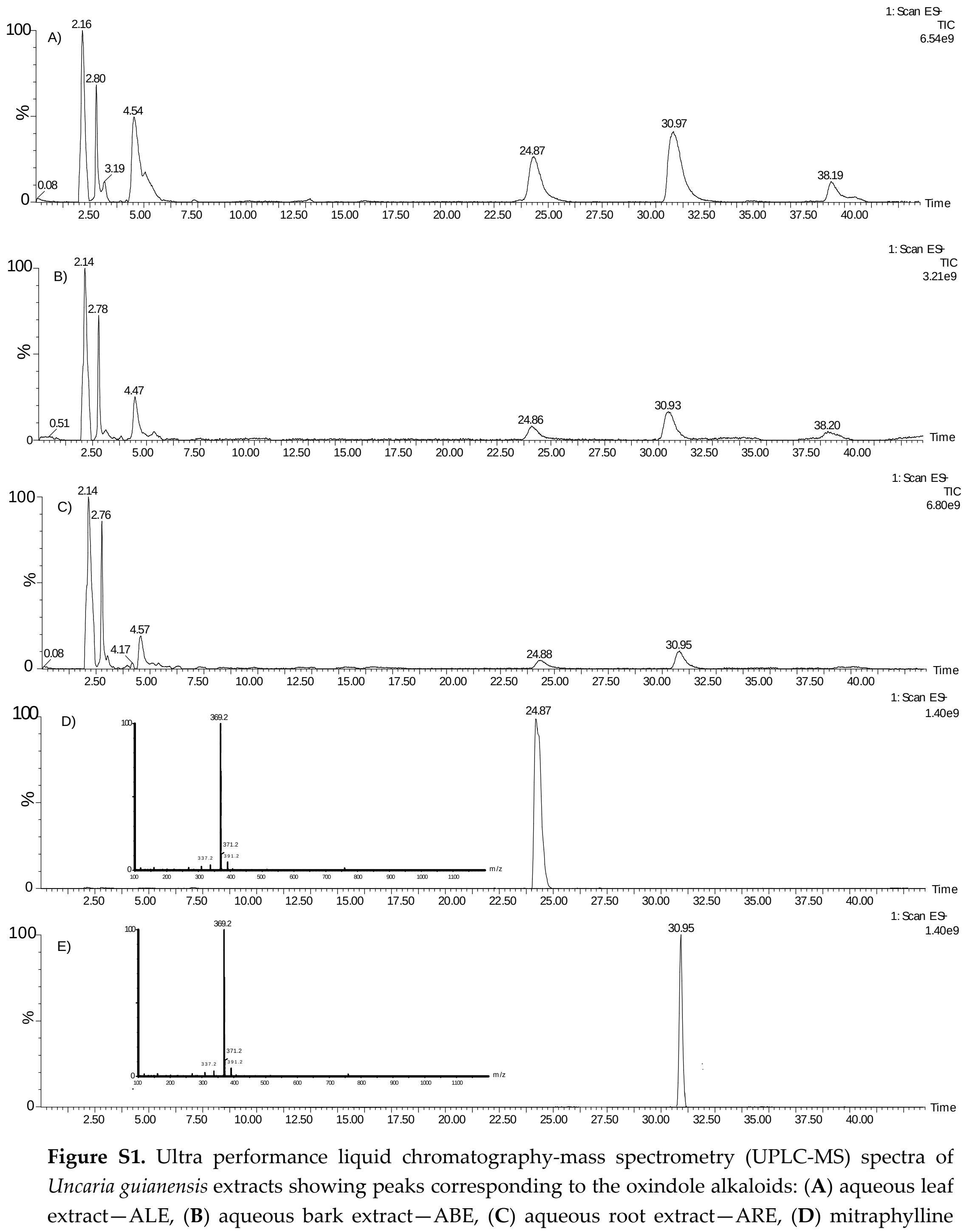

Figure S1. Ultra performance liquid chromatography-mass spectrometry (UPLC-MS) spectra of *Uncaria guianensis* extracts showing peaks corresponding to the oxindole alkaloids: (**A**) aqueous leaf extract—ALE, (**B**) aqueous bark extract—ABE, (**C**) aqueous root extract—ARE, (**D**) mitraphylline standard and (**E**) isomitraphylline standard.

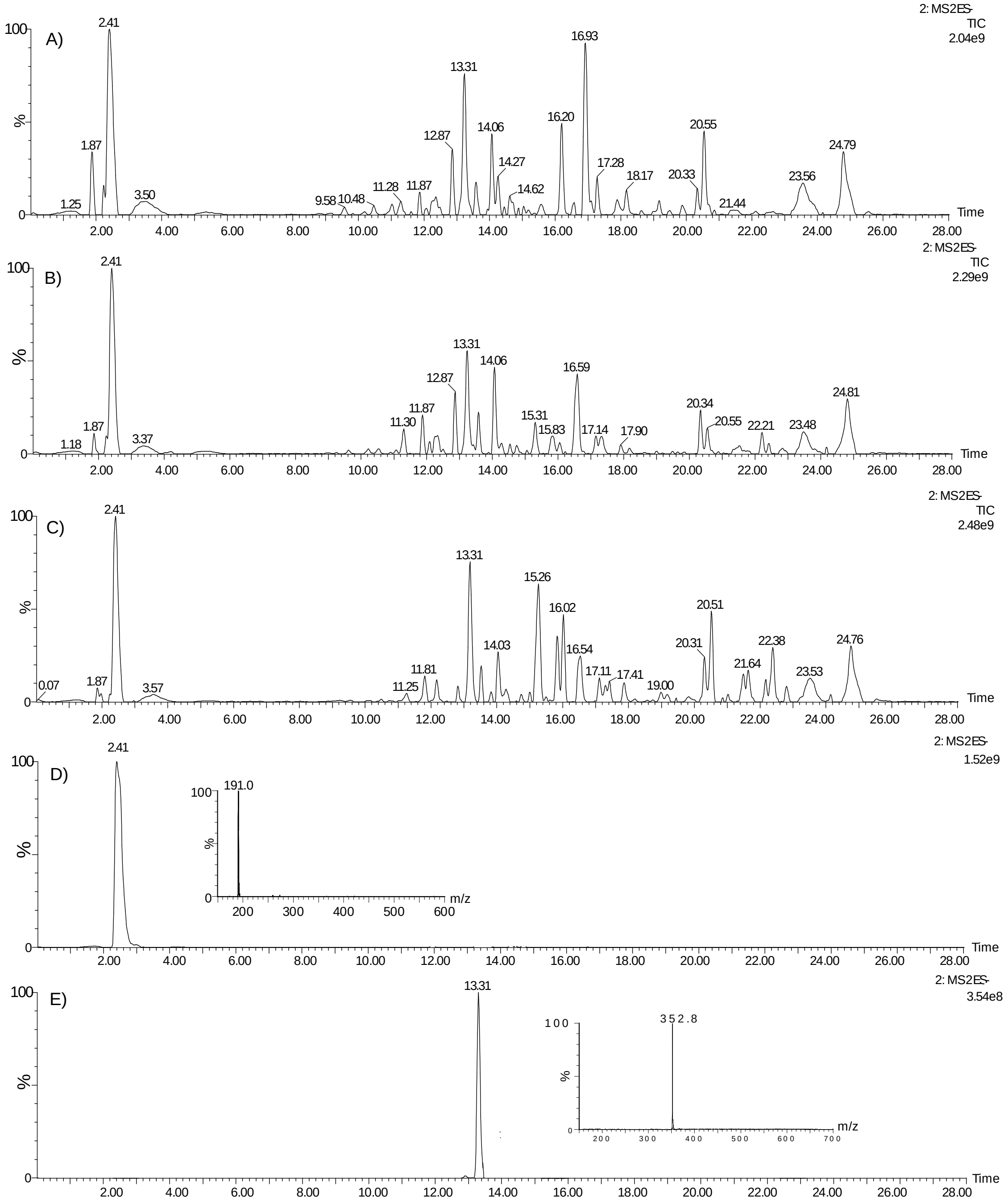

Figure S2. Ultra performance liquid chromatography-mass spectrometry (UPLC-MS) spectra of *Uncaria guianensis* extracts showing peaks corresponding to the phenolic compounds: (**A**) aqueous leaf extract—ALE, (**B**) aqueous bark extract—ABE, (**C**) aqueous root extract—ARE, (**D**) quinic acid standard and (**E**) chlorogenic acid standard.

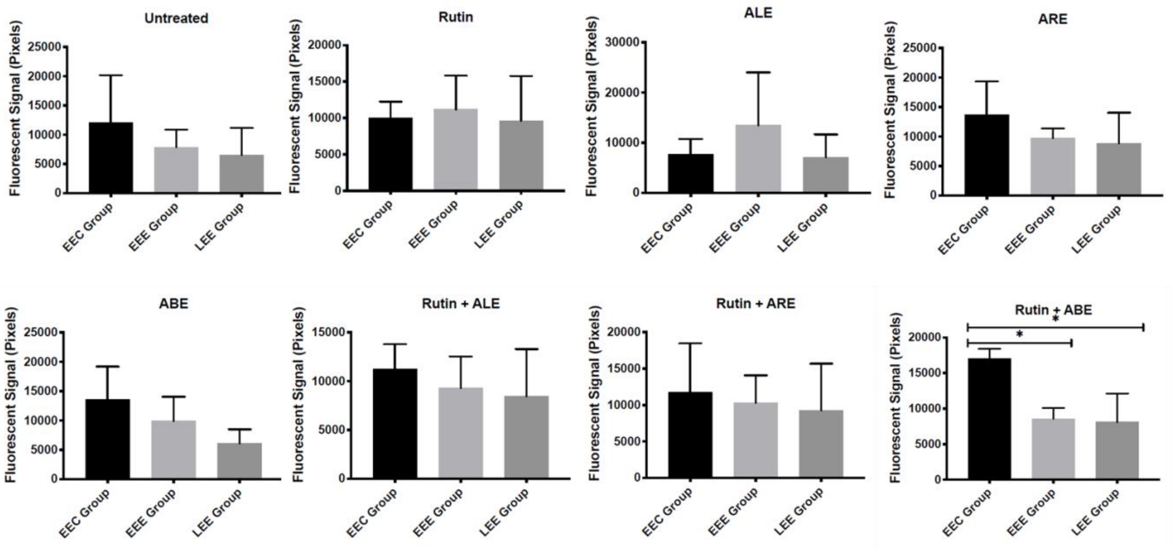

Figure S3. Between-group comparisons showing the intensities of fluorescence of SYTOX™ Green nucleic acid stain indicating dead stromal spheroids isolated from eutopic endometrium of control patients without endometriosis (EEC group), eutopic endometrium of patients with endometriosis/endometrioma (EEE group) and lesions of patients with endometriosis/endometrioma (LEE group). Assays were carried out using a 3D culture technique with untreated spheroids as controls. Treatments comprised rutin, aqueous bark extract (ABE), aqueous leaf extract (ALE) or aqueous root extract (ARE) from *Uncaria guianensis* at 100 μg mL^{-1}, or combinations of rutin + ALE, rutin + ABE and rutin + ARE containing 100 μg mL^{-1} of each component. Each bar represents the mean number of pixels ± standard deviation (n = 3). Data were analyzed by one-way ANOVA and Tukey test except for rutin + ARE in which one-way ANOVA with Welch correction and Dunnett-Tukey T3 multiple comparison test were applied with statistical significance set at $*p < 0.05$.

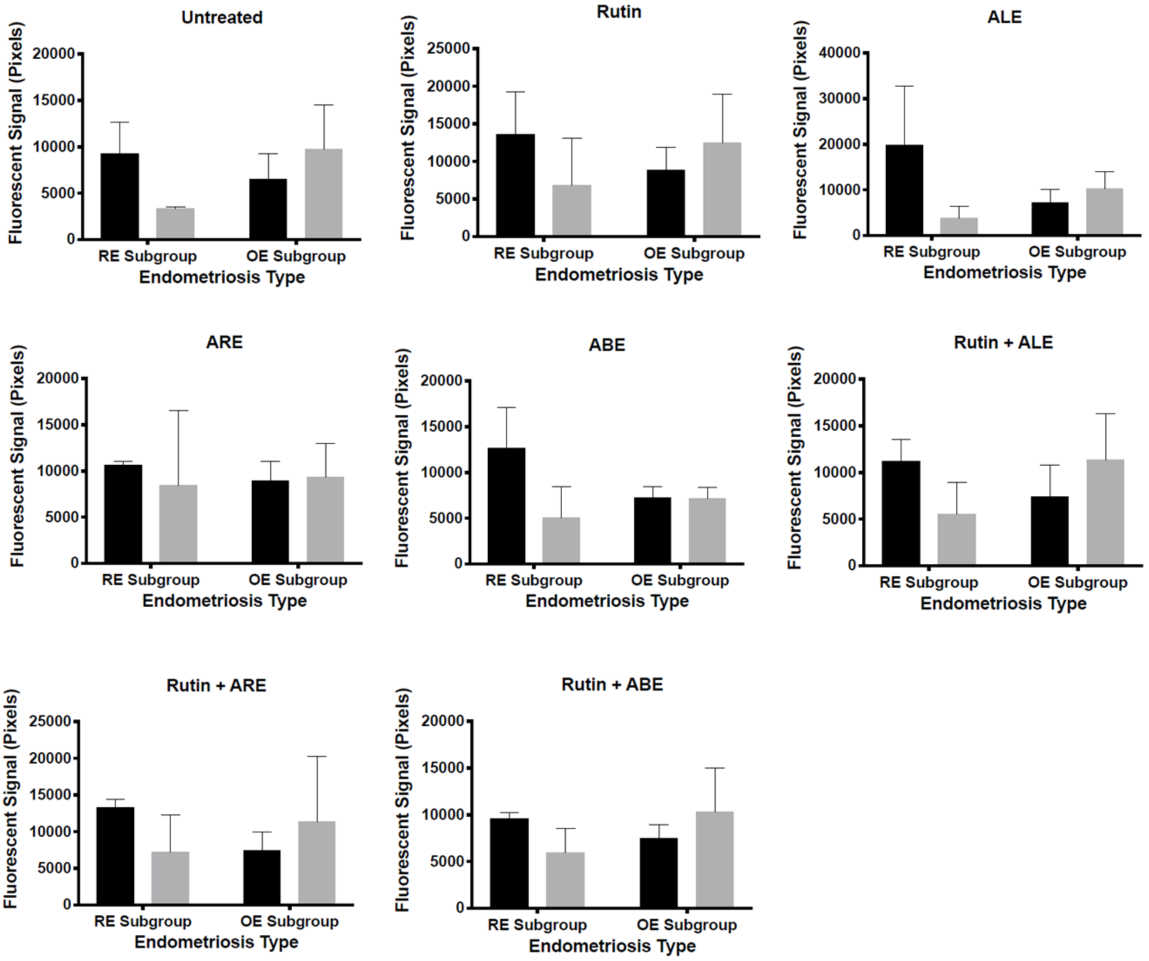

Figure S4. Comparisons of intensities of fluorescence of SYTOX™ Green nucleic acid stain indicating dead stromal spheroids isolated from eutopic endometrium (■) and lesions (■) of patients with retrocervical endometriosis (RE subgroup) and ovarian endometrioma (OE subgroup). Assays were carried out using a 3D culture technique with untreated spheroids as controls. Treatments comprised rutin, aqueous bark extract (ABE), aqueous leaf extract (ALE) or aqueous root extract (ARE) from Uncaria guianensis at 100 µg mL^{-1}, or combinations of rutin + ALE, rutin + ABE and rutin + ARE containing 100 µg mL^{-1} of each component. Each bar represents the mean number of pixels ± standard deviation ($n = 3$). Data were analyzed by two-way ANOVA and Tukey test with statistical significance set at $p < 0.05$.

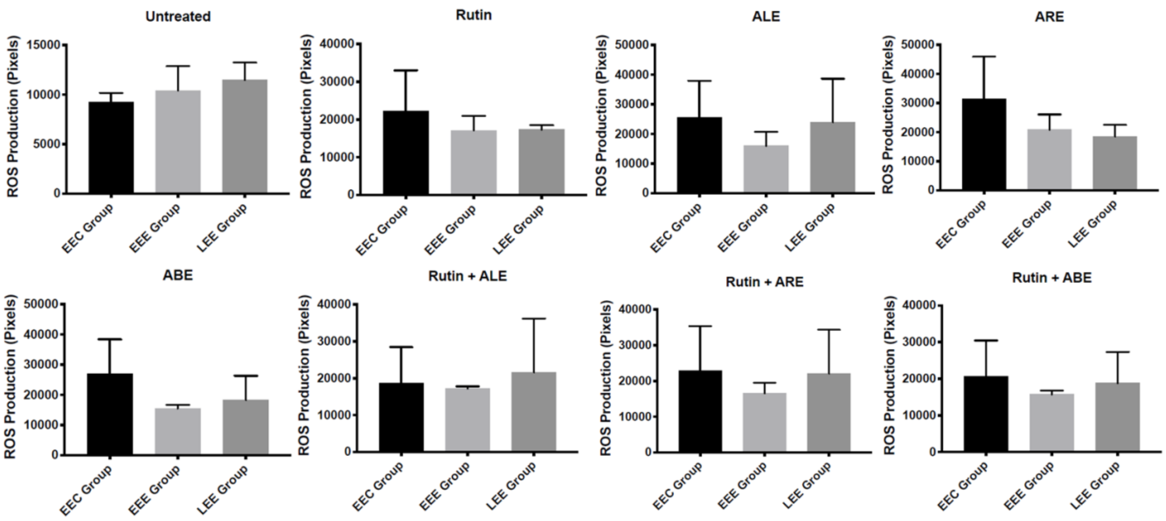

Figure S5. Between-group comparisons showing the intensities of fluorescence of dichlorodihydrofluorescein diacetate probe indicating production of reactive oxygen species (ROS) by stromal cells isolated from eutopic endometrium of control patients without endometriosis (EEC group), eutopic endometrium of patients with endometriosis/endometrioma (EEE group) and lesions of patients with endometriosis/endometrioma (LEE group). Assays were carried out using a 2D culture technique with untreated cells as controls. Treatments comprised rutin, aqueous bark extract (ABE), aqueous leaf extract (ALE) or aqueous root extract (ARE) from Uncaria guianensis at 100 μg mL^{-1}, or combinations of rutin + ALE, rutin + ABE and rutin + ARE containing 100 μg mL^{-1} of each component. Each bar represents the mean number of pixels ± standard deviation (n = 3). Data were analyzed by one-way ANOVA and Tukey test with statistical significance set at $p < 0.05$.

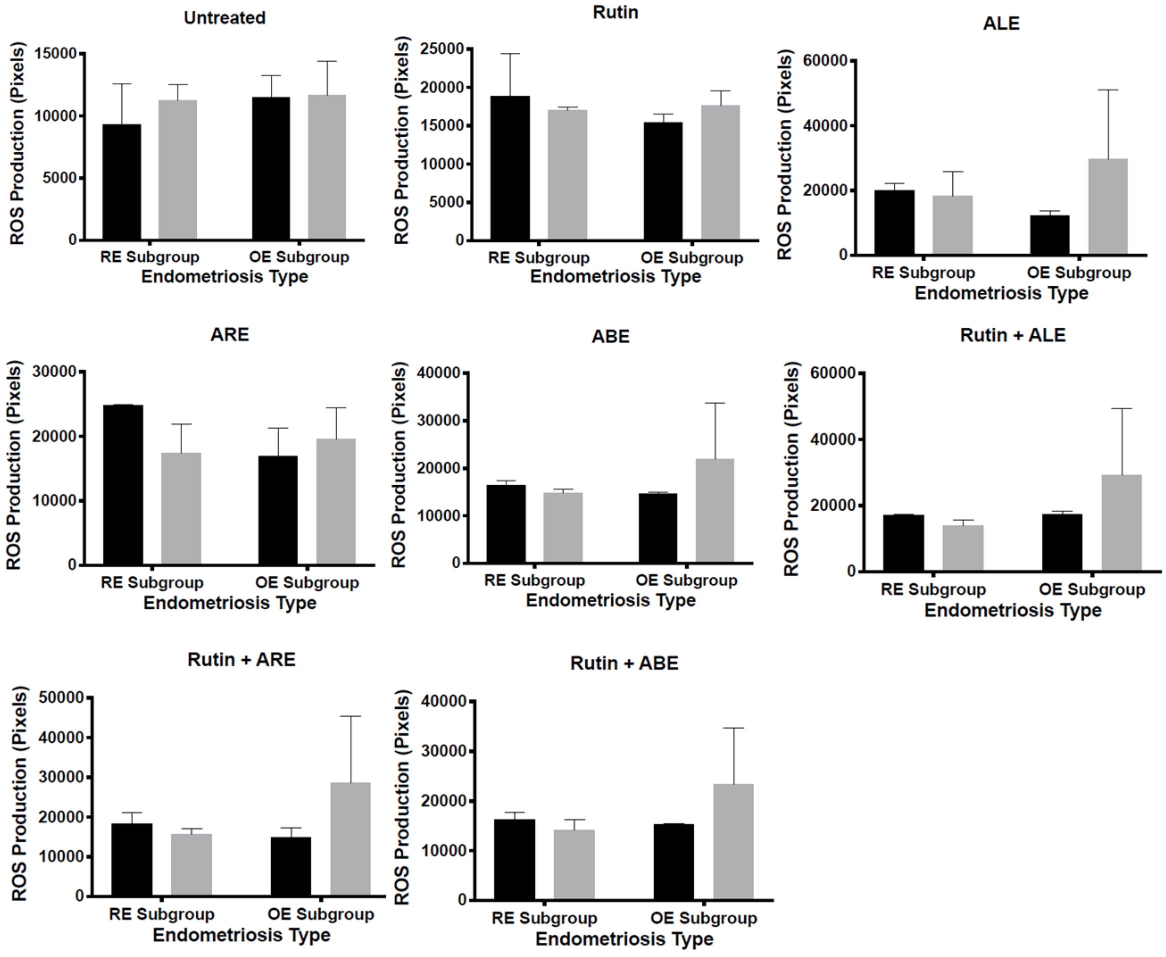

Figure S6. Comparisons of intensities of fluorescence of dichlorodihydrofluorescein diacetate probe indicating production of reactive oxygen species (ROS) by stromal cells isolated from eutopic endometrium (■) and lesions (■) of patients with retrocervical endometriosis (RE subgroup) and ovarian endometrioma (OE subgroup). Assays were carried out using a 2D culture technique with untreated cells as control. Treatments comprised rutin, aqueous bark extract (ABE), aqueous leaf extract (ALE) or aqueous root extract (ARE) from *Uncaria guianensis* at 100 μg mL^{-1}, or combinations of rutin + ALE, rutin + ABE and rutin + ARE containing 100 μg mL^{-1} of each component. Each bar represents the mean number of pixels ± standard deviation ($n = 3$). Data were analyzed by two-way ANOVA and Tukey test with statistical significance set at $p < 0.05$.

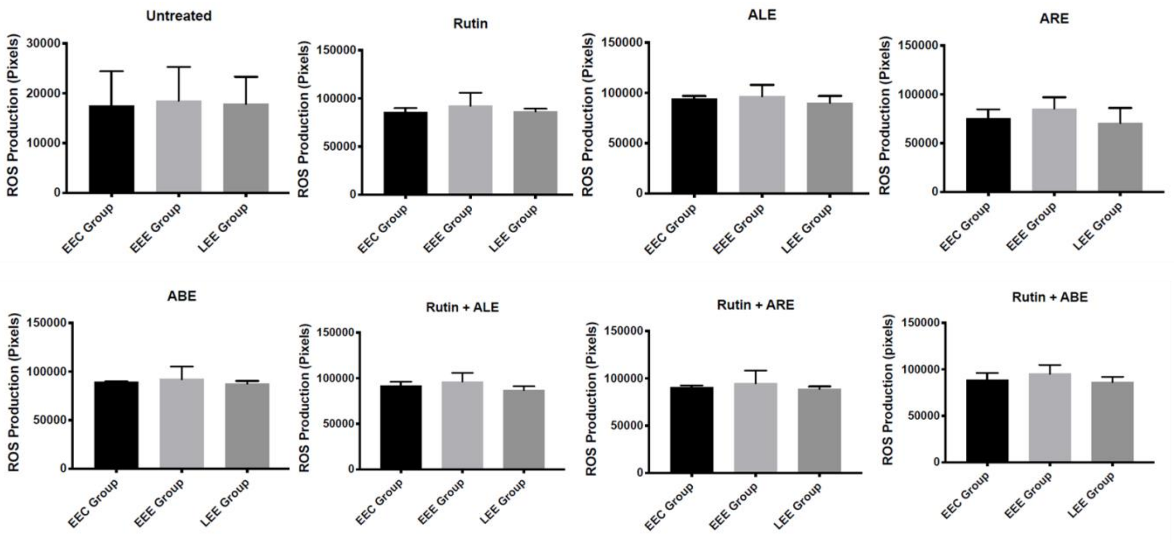

Figure S7. Between-group comparisons showing the intensities of fluorescence of dichlorodihydrofluorescein diacetate probe indicating production of reactive oxygen species (ROS) by stromal spheroids isolated from eutopic endometrium of control patients without endometriosis (EEC group), eutopic endometrium of patients with endometriosis/endometrioma (EEE group) and lesions of patients with endometriosis/endometrioma (LEE group). Assays were carried out using a 3D culture technique with untreated spheroids as controls. Treatments comprised rutin, aqueous bark extract (ABE), aqueous leaf extract (ALE) or aqueous root extract (ARE) from *Uncaria guianensis* at 100 μg mL^{-1}, or combinations of rutin + ALE, rutin + ABE and rutin + ARE containing 100 μg mL^{-1} of each component. Each bar represents the mean number of pixels ± standard deviation ($n = 3$). Data were analyzed by one-way ANOVA and Tukey test with statistical significance set at $p < 0.05$.

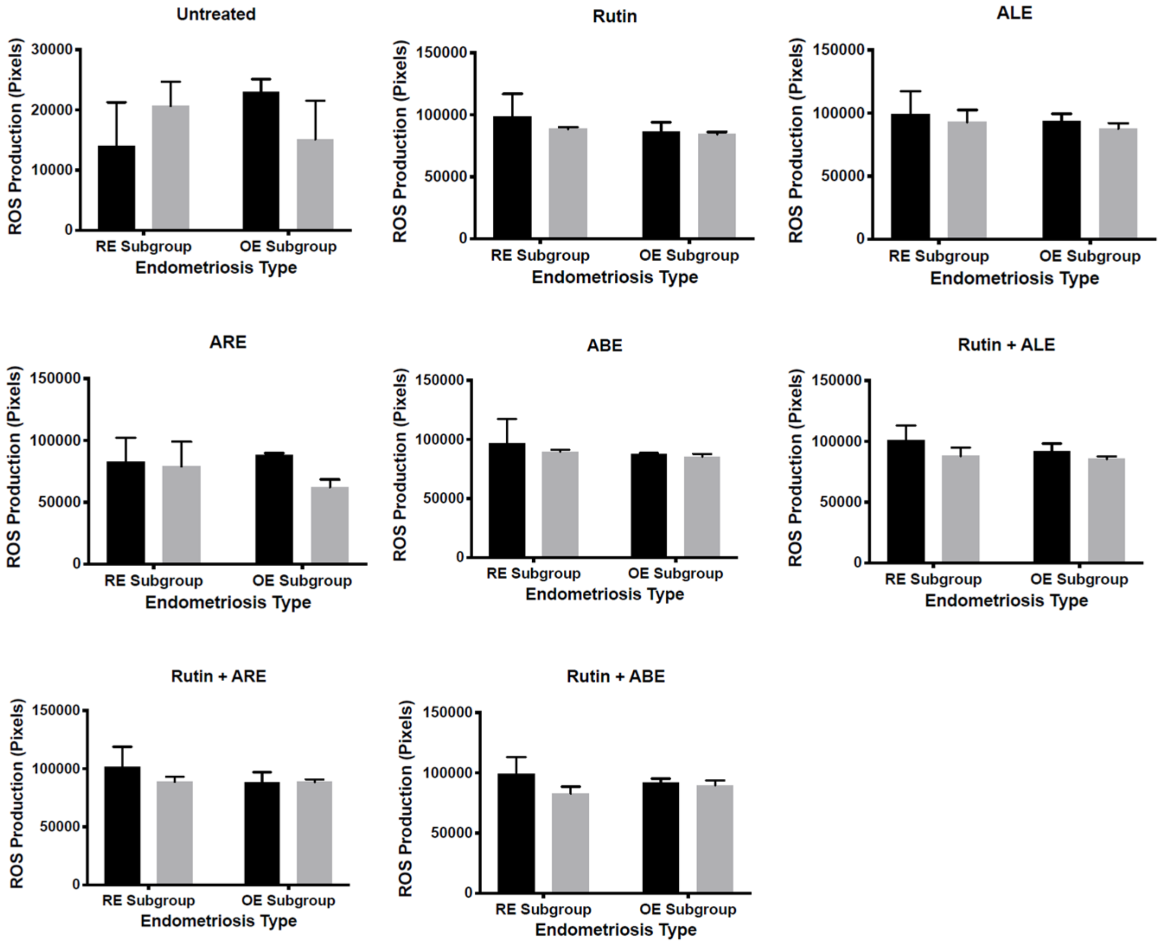

Figure S8. Comparisons of intensities of fluorescence of dichlorodihydrofluorescein diacetate probe indicating production of reactive oxygen species (ROS) by stromal spheroids isolated from eutopic endometrium (■) and lesions (■) of patients with retrocervical endometriosis (RE subgroup) and ovarian endometrioma (OE subgroup). Assays were carried out using a 3D culture technique with untreated spheroids as control. Treatments comprised rutin, aqueous bark extract (ABE), aqueous leaf extract (ALE) or aqueous root extract (ARE) from *Uncaria guianensis* at 100 μg mL^{-1}, or combinations of rutin + ALE, rutin + ABE and rutin + ARE containing 100 μg mL^{-1} of each component. Each bar represents the mean number of pixels ± standard deviation ($n = 3$). Data were analyzed by two-way ANOVA and Tukey test with statistical significance set at $p < 0.05$.

Table S1. Levels of interleukin-15 determined using Milliplex® MAP Human Cytokine/Chemokine Magnetic Bead Panel in 2D cultured stromal cells isolated from eutopic endometrium of patients with endometriosis/endometrioma (EEE group) and treated with aqueous extracts of *Uncaria guianensis* or rutin.

**Treatment µg mL^{-1}**	**Estimated Mean pg mL^{-1} (95% CI)**	**Estimated Mean Difference[1] pg mL^{-1} (95% CI)**	***p* value***
Untreated	0.56 (0.26, 1.23)	-	-
ABE 10	1.03 (0.55, 1.93)	0.47 (−0.20, 1.14)	0.284
**ABE 100**	**1.72 (0.97, 3.06)**	**1.16 (0.04, 2.28)**	**0.038**
ALE 10	1.03 (0.55, 1.93)	0.47 (−0.20, 1.14)	0.284
ALE 100	1.29 (0.71, 2.35)	0.73 (−0.11, 1.57)	0.119
ARE 10	0.92 (0.48, 1.75)	0.36 (−0.20, 0.92)	0.286
ARE 100	1.31 (0.72, 2.38)	0.75 (−0.11, 1.60)	0.119
Rutin 10	0.81 (0.41, 1.57)	0.24 (−0.22, 0.71)	0.293
Rutin 100	1.17 (0.64, 2.14)	0.61 (−0.14, 1.36)	0.166

CI, confidence interval; ABE, aqueous bark extract, ALE, aqueous leaf extract, ARE, aqueous root extract. [1] In relation to untreated cells.* p values were corrected by sequential Bonferroni method. Statistical significance was set at $p < 0.05$.

Table S2. Levels of interleukin-17A determined using Milliplex® MAP Human Cytokine/Chemokine Magnetic Bead Panel in 2D cultured stromal cells isolated from eutopic endometrium of patients with endometriosis/endometrioma (EEE group) and treated with aqueous extracts of *Uncaria guianensis* or rutin.

**Treatment** μg mL^{-1}	**Estimated Mean** pg mL^{-1} (95% CI)	**Estimated Mean Difference**[1] pg mL^{-1} (95% CI)	***p* value***
Untreated	0.14 (−0.10, 0.38)	-	_
ABE 10	0.19 (−0.05, 0.43)	0.05 (−0.20, 0.29)	> 0.999
**ABE 100**	**0.63 (0.39, 0.86)**	**0.48 (0.16, 0.81)**	**0.001**
ALE 10	0.28 (0.04, 0.52)	0.14 (−0.17, 0.44)	> 0.999
ALE 100	0.30 (0.06, 0.54)	0.15 (−0.15, 0.46)	0.995
ARE 10	0.08 (−0.16, 0.32)	−0.06 (−0.31, 0.19)	> 0.999
ARE 100	0.39 (0.15, 0.63)	0.25 (−0.07, 0.56)	0.218
Rutin 10	0.15 (−0.09, 0.39)	0.01 (−0.22, 0.24)	> 0.999
Rutin 100	0.21 (−0.03, 0.45)	0.07 (−0.19, 0.33)	> 0.999

CI, confidence interval; ABE, aqueous bark extract, ALE, aqueous leaf extract, ARE, aqueous root extract. [1] In relation to untreated cells. * p values were corrected by sequential Bonferroni method. Statistical significance was set at $p < 0.05$.

Table S3. Levels of interleukin-4 determined using Milliplex® MAP Human Cytokine/Chemokine Magnetic Bead Panel in 2D cultured stromal cells isolated from eutopic endometrium of patients with endometriosis/endometrioma (EEE group) and treated with aqueous extracts of *Uncaria guianensis* or rutin.

**Treatment** μg mL^{-1}	**Estimated Mean** pg mL^{-1} **(95% CI)**	**Estimated Mean Difference**[1] pg mL^{-1} **(95% CI)**	***p* value***
Untreated	2.71 (0.83, 8.86)	-	-
ABE 10	3.90 (1.62, 9.38)	1.19 (−3.62, 6.01)	> 0.999
**ABE 100**	**13.25 (8.20, 21.41)**	**10.55 (1.65, 19.44)**	**0.013**
ALE 10	8.30 (4.76, 14.47)	5.59 (−0.97, 12.15)	0.128
ALE 100	8.81 (5.12, 15.16)	6.11 (−0.79, 13.00)	0.107
ARE 10	3.39 (1.27, 9.04)	0.69 (−3.86, 5.23)	> 0.999
ARE 100	9.12 (5.33, 15.58)	6.41 (−0.73, 13.55)	0.101
Rutin	4.60 (2.11, 10.00)	1.89 (−3.37, 7.15)	> 0.999
Rutin	4.79 (2.25, 10.20)	2.09 (−3.57, 7.74)	> 0.999

CI, confidence interval; ABE, aqueous bark extract, ALE, aqueous leaf extract, ARE, aqueous root extract. [1] In relation to untreated cells. * p values were corrected by sequential Bonferroni method. Statistical significance was set at $p < 0.05$.

Table S4. Levels of interleukin-6 determined using Milliplex® MAP Human Cytokine/Chemokine Magnetic Bead Panel in 2D cultured stromal cells isolated from eutopic endometrium of patients with endometriosis/endometrioma (EEE group) and treated with aqueous extracts of *Uncaria guianensis* or rutin.

**Treatment** μg mL^{-1}	**Estimated Mean** pg mL^{-1} **(95% CI)**	**Estimated Mean Difference**[1] pg mL^{-1} **(95% CI)**	***p* value***
Untreated	62.21 (−4.17, 128.58)	-	-
ABE 10	78.81 (12.44, 145.18)	16.60 (−36.93, 70.14)	> 0.999
**ABE 100**	**161.68 (95.31, 228.05)**	**99.48 (43.71, 155.25)**	**< 0.001**
ALE 10	76.66 (10.29, 143.03)	14.45 (−31.09, 60.00)	> 0.999
ALE 100	67.17 (0.80, 133.54)	4.97 (−35.68, 45.62)	> 0.999
ARE 10	75.51 (9.14, 141.88)	13.30 (−31.58, 58.19)	> 0.999
ARE 100	113.47 (47.09, 179.84)	51.26 (−3.48, 106.00)	0.078
Rutin 10	64.58 (−1.79, 130.95)	2.37 (−37.16, 41.90)	> 0.999
Rutin 100	65.90 (−0.47, 132.27)	3.69 (−36.39, 43.78)	> 0.999

CI, confidence interval; ABE, aqueous bark extract, ALE, aqueous leaf extract, ARE, aqueous root extract. [1] In relation to untreated cells. * p values were corrected by sequential Bonferroni method. Statistical significance was set at $p < 0.05$.

Table S5. Levels of tumor necrosis factor-alpha determined using Milliplex® MAP Human Cytokine/Chemokine Magnetic Bead Panel in 2D cultured stromal cells isolated from eutopic endometrium of patients with endometriosis/endometrioma (EEE group) and treated with aqueous extracts of *Uncaria guianensis* or rutin.

**Treatment** **μg mL^{-1}**	**Estimated Mean** **pg mL^{-1} (95% CI)**	**Estimated Mean Difference**[1] **pg mL^{-1} (95% CI)**	***p* value***
Untreated	1.00 (−1.06, 3.06)	-	-
ABE 10	1.40 (−0.66, 3.46)	0.40 (−1.47, 2.27)	>0.999
**ABE 100**	**3.75 (1.69, 5.81)**	**2.75 (0.29, 5.20)**	**0.021**
ALE 10	1.84 (−0.22, 3.90)	0.84 (−1.29, 2.96)	>0.999
ALE 100	1.11 (−0.95, 3.17)	0.10 (−1.63, 1.84)	>0.999
ARE 10	1.12 (−0.94, 3.18)	0.12 (−1.63, 1.86)	>0.999
ARE 100	2.18 (0.11, 4.24)	1.17 (−1.23, 3.58)	>0.999
Rutin 10	1.01 (−1.06, 3.07)	0.00 (−1.70, 1.70)	>0.999
Rutin 100	1.53 (−0.53, 3.59)	0.52 (−1.42, 2.47)	>0.999

CI, confidence interval; ABE, aqueous bark extract, ALE, aqueous leaf extract, ARE, aqueous root extract. [1] In relation to untreated cells. * p values were corrected by sequential Bonferroni method. Statistical significance was set at $p < 0.05$.

Table S6. Levels of vascular endothelium growth factor determined using Milliplex® MAP Human Cytokine/Chemokine Magnetic Bead Panel in 2D cultured stromal cells isolated from eutopic endometrium of patients with endometriosis/endometrioma (EEE group) and treated with aqueous extracts of *Uncaria guianensis* or rutin.

**Treatment** µg mL^{-1}	**Estimated Mean** pg mL^{-1} (95% CI)	**Estimated Mean Difference**[1] pg mL^{-1} (95% CI)	*p* **value***
Untreated	0.00 (−4.55, 4.55)	-	-
ABE 10	0.48 (−4.07, 5.03)	0.48 (−5.32, 6.28)	> 0.999
**ABE 100**	**11.08 (6.53, 15.63)**	**11.08 (2.99, 19.18)**	**0.003**
ALE 10	0.48 (−4.07, 5.03)	0.48 (−5.32, 6.28)	> 0.999
ALE 100	6.42 (1.86, 10.97)	6.41 (−1.36, 14.19)	0.158
ARE 10	1.28 (−3.27, 5.83)	1.28 (−4.89, 7.45)	> 0.999
ARE 100	7.52 (2.97, 12.07)	7.52 (−0.43, 15.46)	0.073
Rutin 10	2.04 (−2.51, 6.59)	2.04 (−5.53, 9.60)	> 0.999
Rutin 100	0.82 (−3.74, 5.37)	0.81 (−5.13, 6.76)	> 0.999

CI, confidence interval; ABE, aqueous bark extract, ALE, aqueous leaf extract, ARE, aqueous root extract. [1] In relation to untreated cells. * p values were corrected by sequential Bonferroni method. Statistical significance was set at $p < 0.05$.

Table S7. Levels of epidermal growth factor determined using Milliplex® MAP Human Cytokine/Chemokine Magnetic Bead Panel in 2D cultured stromal cells isolated from eutopic endometrium of patients with endometriosis/endometrioma (EEE group) and treated with aqueous extracts of *Uncaria guianensis* or rutin.

**Treatment** µg mL^{-1}	**Estimated Mean** pg mL^{-1} (95% CI)	**Estimated Mean Difference[1]** pg mL^{-1} (95% CI)	***p* value***
Untreated	3.52 (1.52, 5.53)	-	-
ABE 10	2.64 (0.48, 4.79)	−0.89 (−3.36, 1.58)	> 0.999
ABE 100	4.29 (2.13, 6.45)	0.77 (−1.63, 3.17)	> 0.999
ALE 10	1.75 (−0.25, 3.75)	−1.77 (−4.36, 0.81)	0.364
**ALE 100**	**7.07 (5.06, 9.07)**	**3.54 (0.85, 6.24)**	**0.005**
ARE 10	2.66 (0.65, 4.66)	−0.87 (−3.30, 1.56)	>0.999
ARE 100	4.96 (2.95, 6.96)	1.43 (−1.08, 3.95)	0.625
Rutin 10	3.03 (1.03, 5.03)	−0.49 (−2.58, 1.59)	>0.999
Rutin 100	1.67 (−0.33, 3.68)	−1.85 (−4.49, 0.79)	0.357

CI, confidence interval; ABE, aqueous bark extract, ALE, aqueous leaf extract, ARE, aqueous root extract. [1] In relation to untreated cells. * p values were corrected by sequential Bonferroni method. Statistical significance was set at $p < 0.05$.
